# The Treatment of Salpingitis by Depletion and Drainage, Secured by Electricity

**Published:** 1892-12

**Authors:** Augustin H. Goelet

**Affiliations:** New York


### THE TREATMENT OF SALPINGITIS BY DEPLETION AND DRAINAGE, SECURED BY ELECTRICITY.*

By AUGUSTIN H. GOELET, M. D.,

NEW YORK.

The author stated that he had recently reported at the New York Obstetrical Society, a number of cases treated successfully by this method, but some of the members were inclined to doubt the part played by electricity in producing the results, and objections were made to electro-therapeutics in general, on the ground that it was made necessarily mysterious and complicated. They endeavored to explain the results obtained by supposing that they were due to rest in bed, and the time consumed in the treatment. In reply to these criticisms, the author said that the patients were not confined to bed, and the treatment was insti-

*Abstract of paper read before the American Electro-Therapeutic Association, October 5, 1892.

tuted after protracted efforts by other methods in the hands of other specialists, and after laparotomy had been advised.

He thought that if these gentlemen, who found so much mysticism surrounding the subject, would study electricity, learn the principle of its action, and take the trouble to understand it, the mysticism would disappear. One gentleman thought hot water would do all that had been claimed by the author for electricity, and that it would relieve pain as promptly, in spite of the fact that in the cases reported all the usual remedies, including hot water, had been tried with marked benefit.

Dr. Goelet was not inclined to deny the value of hot water and other time-honored measures, nor question their usefulness, but that they, at times fail, are slow in their action, and are limited in their usefulness, he thought would not be denied.

The author contended that the principles involved in the electrical treatment were the same as in the other measures usually adopted for the treatment of these conditions, namely, depletion and drainage, and with perhaps stimulation of the lymphatics to promote absorption. But he did not believe, as some had contended, that counter-irritation entered into the action of the electrical treatment. He was inclined to favor the method of dilatation, currettement, and drainage with gauze in certain conditions, but thought its usefulness was limited, and electricity could be employed in conditions where this would not be appropriate. He was inclined to believe that electricity was not more universally employed, because its value was not appreciated, and less pains was taken to better understand it; and in consequence, it was regarded as mysterious, and its results were discredited, because it was considered too intricate to be clearly appreciated without some thought and study.

The author explained his preference for electricity in these conditions by saying that after an experience of fifteen years with the older methods of treatment, he had found them tedious, slow, and exceedingly unsatisfactory, and was willing to welcome any plan which promised more prompt and satisfactory results, and which saved the patient from a mutilating operation, the results of which are, by no means, always what could be desired.

He frankly confessed that the method of treatment advocated by him had given both him and his patients more satisfaction than any other, and he believed that the more one used it, the better he knew how to use it, and the better the results. He would not say, however, that this particular method was appropriate in all conditions, or that it should be used always to the exclusion of other remedies; and declared that he had used the method suggested by Dr. Polk and others, with satisfaction in suitable cases.

The author thought it would hardly seem necessary to say that, in order to obtain satisfactory results with this or any other therapeutic measure, it should be used intelligently and with a definite purpose in view, yet electricity is frequently used in a haphazard manner by men who wonder why they do not succeed; and their failures cause them to discredit the value of the agent. He regarded two things essential for success, namely: first, an accurate diagnosis of the existing condition with the indications for treatment; and second, the employment of the agent for a definite purpose, and in a manner to meet the indications. He employs the galvanic current for the purpose of effecting relaxation and dilatation of the cervical canal with drainage from the uterine cavity, which is accomplished by using the negative pole attached to a metallic electrode placed in the canal. A moderate strength of current is employed, when dilatation without cauterization is desired; but an increased strength is necessary when destruction of the diseased endometrium is required. Profuse drainage, resulting in depletion, follows the use of this pole employed in this manner, and relaxation with dilatation of the lumen of the Fallopian tubes when it is obstructed by tumefaction of the mucous lining, likewise results from the use of this pole in the uterus. He quoted Bland Sutton as saying that the uterus end of the tube is really occluded in salpingitis, but the obstruction is due to tumefaction of the mucous lining; hence drainage in this direction, is possible only upon subsidence of this tumefaction. The same authority declares that the tender pelvic swellings found in these conditions, are in many instances the Fallopian tubes swollen in consequence of the catarrhal condition of the mucous membrane, and that the swelling, pain and tenderness vanish with the subsidence of the inflammation.

Dr. Goelet thought that the accomplishment of this result here should not be considered unreasonable, when it is possible in other localities. For this purpose, he was in the habit of employing both the galvanic current, as above described, and the faradic current, also, for the relief of the associated pain and congestion. The intra-uterine applications likewise affect the cure of the endometritis which, in all probability, was the origin of the tubal inflammation and is directly responsible for its continuance. He thought the positive pole was contraindicated if there was any pus accumulation, or if there was insufficient drainage from the tube, but employed it in the absence of these contraindications to aid the removal of tumefaction and congestion, employing the vagino-abdominal method by preference.

In commencing the treatment of these cases, he prefers to employ the faradic current of tension, first to quiet the pain and render the patient comfortable, for which purpose he utilizes the bi-polar method in the vagina; later, he continues the use of the faradic applications to the vagina for the purpose of stimulating peristaltic movements of the tubes which favor evacuation. He drew attention to the fact that, in order to obtain the desired effect from the faradic current it was essential to utilize reliable apparatus, otherwise failure, rather than success, would follow its use.

In conclusion, he remarked that the chief indications in the treatment of these conditions, was to relieve the pain and remove the engorgement and tumefaction and to secure drainage from the uterine cavity, and the tubes, and he believed that electricity, if properly employed, could be depended upon to accomplish these results.

---

Hemorrhoids rapidly diminish in size, become free from secretion and pain, and generally improve, according to Dr James, if powdered daily with pure calomel.—*New York Medical Record.*

